# MicroRNA-23b functions as an oncogene and activates AKT/GSK3*β*/*β*-catenin signaling by targeting ST7L in hepatocellular carcinoma

**DOI:** 10.1038/cddis.2017.216

**Published:** 2017-05-18

**Authors:** Likun Zhuang, Xin Wang, Zusen Wang, Xiang Ma, Bing Han, Hao Zou, Zehua Wu, Sheng Dong, Zhiqiang Qu, Yunjin Zang, Liqun Wu

**Affiliations:** 1 Institute of Transplantation Science, The Affiliated Hospital of Qingdao University, Qingdao, China; 2 Department of Hepatobiliary and Pancreatic Surgery, The Affiliated Hospital of Qingdao University, Qingdao, China; 3 Center for Medical Research, The Affiliated Hospital of Qingdao University, Qingdao, China

## Abstract

Hepatocellular carcinoma (HCC) is a malignant tumor and threatens human life worldwide, whereas the etiology and pathogenesis of HCC have not been fully determined. In the past few years, many microRNAs (miRNAs) have been proved to have important roles in tumorigenesis of HCC. In this study, we found that miR-23b was significantly upregulated in tumor tissues of HCC patients. Functional tests showed that miR-23b could promote HCC cell proliferation and metastasis *in vitro* and *in vivo*. Then, mechanistic investigations suggested that ST7L was a direct target of miR-23b and involved in the promotion effects of miR-23b on HCC tumorigenesis and metastasis. Furthermore, our study indicated that ST7L could interact with the carboxyl terminal region of AKT and suppress AKT/GSK3*β*/*β*-catenin pathway in HCC cells. In conclusion, our study revealed important roles of miR-23b and ST7L in progression of HCC.

Hepatocellular carcinoma (HCC) is a severe malignancy with high mortality worldwide. Until now, liver resection and transplantation are the most common treatments for HCC patients.^1^ Despite improved therapies during the past decades, HCC remains one of the leading causes of cancer-related deaths for its recurrence and metastasis. To compromise this effect, many studies are devoted to exploring the molecular pathogenesis of HCC.

MicroRNAs (miRNAs) are small non-coding RNA molecules (18–25 nucleotides), which could bind to the 3′-untranslated region (3′-UTR) of target mRNA and silence the expression levels of target genes.^2^ Many reports showed that miRNAs could serve as oncogenes or tumor-suppressor genes in tumorigenesis.^3^ MiRNAs were also reported to have important roles in multiple biological processes during HCC occurrence and progression. For instance, our previous study showed that miR-92b could promote cell proliferation and metastasis of HCC by suppressing the expression of tumor-suppressor gene Smad7.^4^ Another study reported that miR-28-5p directly targeted IL-34 and had important roles in HCC growth and metastasis.^5^ Although many miRNAs were indicated to be related with pathogenesis of HCC, the roles of the majority of miRNAs in HCC remain to be explored. MiRNAs have been considered as potential diagnostic biomarkers and therapy targets for HCC, and the relationship between miRNAs and HCC is still a hot topic.

We conducted a high-throughput sequencing of small RNAs and found that miR-23b was upregulated in HCC tissues compared with the paired adjacent non-tumor liver tissues (ANLTs). MiR-23b was indicated to affect various biological functions including tumor growth, angiogenesis and metastasis in prostate cancer, breast cancer and gastric carcinoma by repressing a series of target genes.^6, 7, 8^ Recently, miR-23b was reported to suppress activation of hepatic stellate cells by targeting gremlin1 and had a pivotal role in the process of hepatic fibrosis.^9^ However, until now, the roles of miR-23b in HCC progression and the mechanism underlying the effects of miR-23b on HCC tumorigenesis are still needed to be explored.

In the past few years, activation of AKT/GSK3*β*/*β*-catenin pathway was reported to be implicated in tumorigenesis of HCC.^10^ AKT was phosphorylated on Thr308 in the catalytic domain and Ser473 in the C-terminal domain by PDK1 and 2, respectively.^11, 12^ Then, phosphorylated AKT in turn increased GSK3*β* phosphorylation at Ser9, which could inhibit *β*-catenin phosphorylation and degradation by proteasomal pathway.^13^ *β*-Catenin increased expression levels of a series of downstream oncogenes. AKT/GSK3*β*/*β*-catenin pathway mediated a wide variety of biological responses including cell proliferation and metastasis. It seems that AKT/GSK3*β*/*β*-catenin pathway may be a potential treatment target for HCC. ST7L was reported to inhibit the *β*-catenin signaling pathway and act as a tumor-suppressor gene in many cancers.^14, 15^ However, the significance of ST7L in HCC progression has not been elucidated.

In this study, we investigated the roles of miR-23b in HCC progression *in vitro* and *in vivo*. In addition, we determined that ST7L was a target gene of miR-23b and was involved in the effects of miR-23b on HCC progression. Furthermore, mechanistic investigation showed that ST7L could interact with C-terminal domain of AKT and inhibit the activation of AKT/GSK3*β*/*β*-catenin pathway in HCC cells. Our findings provided new insights into the molecular function of miR-23b, ST7L and AKT signaling pathway in tumorigenesis of HCC.

## Results

### MiR-23b is upregulated in tumor tissues of HCC

**Figure 1:**
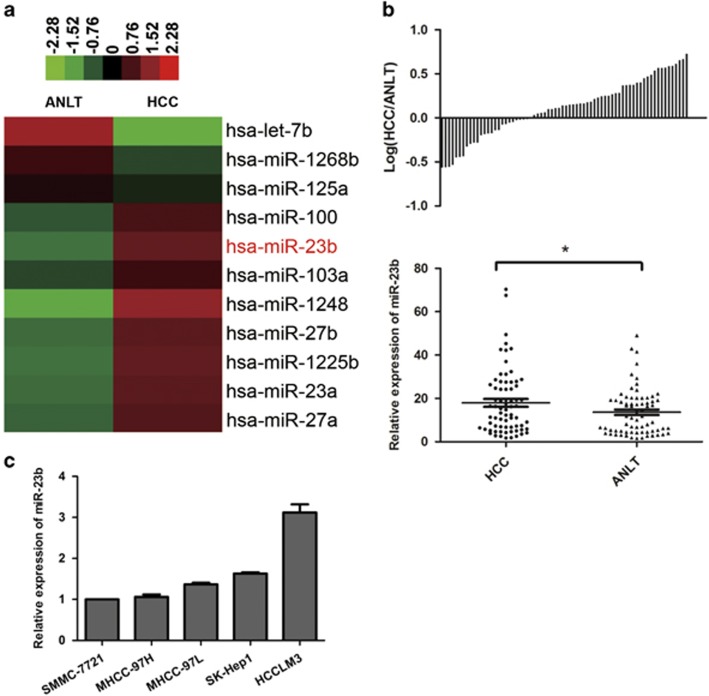
Expression levels of miR-23b in HCC tissues and cell lines. (**a**) Partial miRNA expression profiles of HCC tissues and ANLTs. Red or green color in heatmap separately indicates high or low expression according to the color bar in logarithmic scale shown above the heatmap. (**b**) Expression levels of miR-23b in 70 pairs of HCC tissues and ANLTs were determined by qRT-PCR and normalized to RNU66 expression. Upper panel: the bars represented the relative miR-23b expression with the ratio of its level in HCC tissue *versus* that in ANLT in logarithmic scale. Lower panel: miR-23b expression levels in HCC tissues and ANLTs were compared with paired Student’s *t*-test. (**c**) Relative miR-23b expression levels in HCC cell lines were determined by qRT-PCR. The data were normalized to the expression level of miR-23b in SMMC-7721 cells. Results were represented as mean±S.D. (*n*=3). *P<0.05

First, we screened differential miRNA expression levels between HCC and paired ANLTs by small RNA high-throughput sequencing. The results showed that miR-23b, which mediated tumorigenesis of many cancers, was one of the upregulated miRNAs in HCC tissues (Figure 1a). Then, we measured expression levels of miR-23b in 70 pairs of HCC tissues and ANLTs by quantitative reverse transcription-PCR (qRT-PCR). Consistent with the results of high-throughput sequencing, the expression levels of miR-23b in HCC tissues were significantly higher than that in ANLTs (Figure 1b). Next, we evaluated levels of miR-23b in HCC cell lines. Our results showed that HCCLM3 cells with the highest potential of metastasis expressed the highest miR-23b expression (Figure 1c).

Furthermore, we explored the relationship between miR-23b expression and clinicopathological data. MiR-23b expression levels in HCC tissues were divided into two groups including the high expression group (higher than that in corresponding ANLTs) and the low expression group (lower than that in corresponding ANLTs). The results showed that high miR-23b expression was related to large tumor size, multiple tumors and advanced BCLC stage (Table 1), which suggested that miR-23b level was correlated with HCC progression.

### MiR-23b promotes proliferation, colony formation and metastasis of HCC cells *in vitro*

**Figure 2:**
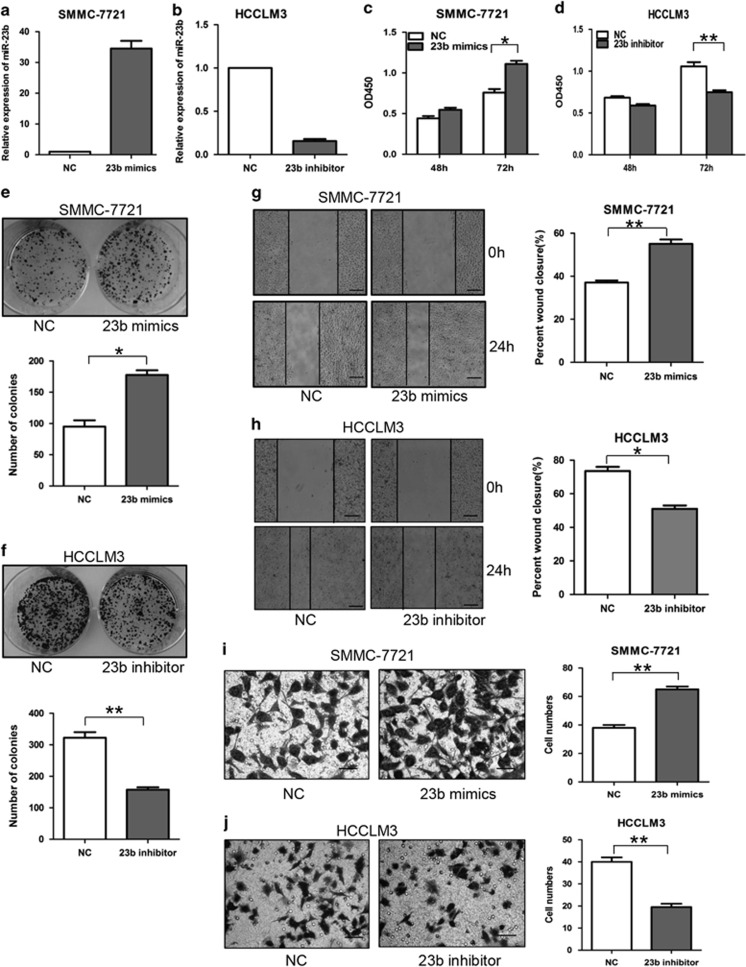
Effects of miR-23b on HCC cell proliferation, colony formation and metastasis *in vitro*. (**a** and **b**) SMMC-7721 cells were transiently transfected with miR-23b mimics and HCCLM3 cells were transfected with miR-23b inhibitor. (**c** and **d**) CCK-8 analysis, (**e** and **f**) colony formation assays, (**g** and **h**) wound-healing assays and (**i** and **j**) transwell invasion assays were conducted. The number of cell colonies was counted and compared in the diagrams below. The percentage of wound closure and the number of cells passed through the membrane were counted and compared in the right diagrams. Scale bars represent 500 *μ*m (**g** and **h**) and 200 *μ*m (**i** and **j**). Results were represented as mean±S.D. (*n*=3). *P<0.05, **P<0.01

In order to investigate the biological function of miR-23b in HCC cells, we upregulated miR-23b expression in SMMC-7721 cells and silenced it in HCCLM3 cells (Figures 2a and b). Cell counting kit-8 (CCK-8) assays showed that upregulating miR-23b promoted cell proliferation of SMMC-7721 cells, whereas downregulating miR-23b significantly inhibited cell proliferation of HCCLM3 cells (Figures 2c and d). Compared with the control cells, the number of cell colonies formed was increased when SMMC-7721 cells were transfected with miR-23b mimics and decreased when HCCLM3 cells were transfected with miR-23b inhibitor (Figures 2e and f). Wound-healing and transwell assays indicated that overexpression of miR-23b promoted migration and invasion of SMMC-7721 cells, whereas knockdown of miR-23b could inhibit migration and invasion of HCCLM3 cells (Figures 2g and j). These data together suggested that miR-23b was able to promote the proliferation and metastasis of HCC cells *in vitro*.

### ST7L is a direct target of miR-23b

**Figure 3:**
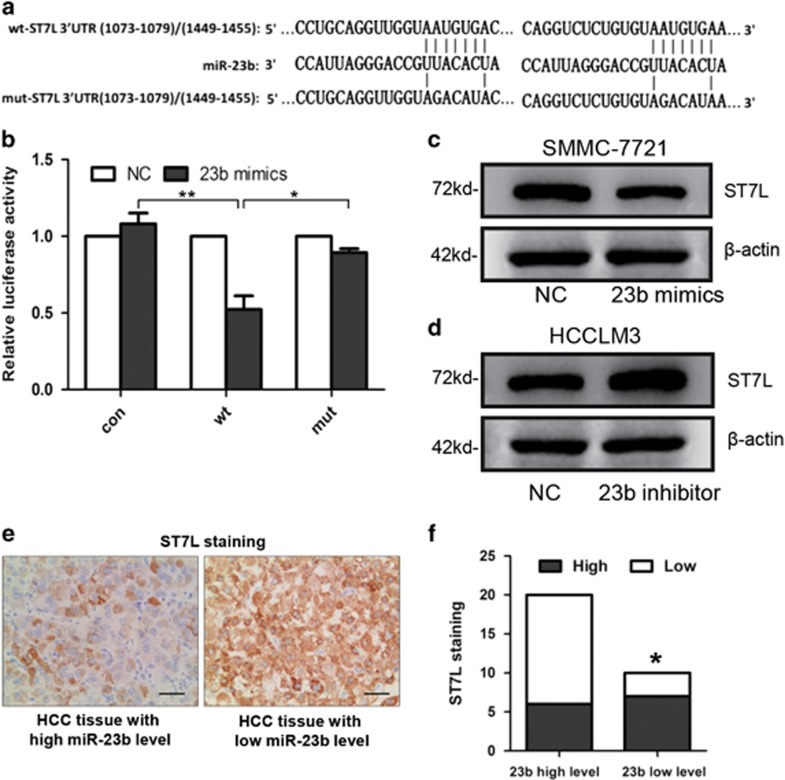
ST7L is a direct target of miR-23b in HCC. (**a**) Diagram of predicted binding sites and corresponding mutant sites in the 3′-UTR of ST7L mRNA (wt, wild-type; mut, mutant type). (**b**) Effects of miR-23b on the expression of ST7L 3′-UTR-containing reporter genes. Luciferase activity was normalized to the value obtained in the cells transfected with NC mimics. Results were represented as mean±S.D. (*n*=3). (**c** and **d**) Western blot analysis of ST7L expression in SMMC-7721 cells transiently transfected with miR-23b mimics and HCCLM3 cells transfected with miR-23b inhibitor. (**e**) Representative immunohistochemistry images showing the expression of ST7L (brown color) in HCC tissues with high miR-23b expression (left) or low miR-23b expression (right). (**f**) A significant inverse correlation between miR-23b and ST7L expression in HCC tissues (Spearman's r=−0.3805). *P<0.05, **P<0.01

Via Targetscan and microRNA.org, we focused on ST7L, which was known to inhibit cell proliferation and invasion of cancer cells, as a potential target gene for miR-23b. There were two predicted miR-23b-binding sites in the 3′-UTR of ST7L mRNA. First, we constructed reporter gene plasmids with ST7L 3′-UTR-containing wild-type (wt) or mutant (mut) miR-23b binding sites (Figure 3a). Luciferase assay indicated that miR-23b suppressed the expression of reporter gene carrying wt 3′-UTR but not that containing mut 3′-UTR (Figure 3b). Then, transfection of miR-23b mimics significantly inhibited ST7L expression in SMMC-7721 cells (Figure 3c), whereas knockdown of miR-23b increased ST7L expression in HCCLM3 cells (Figure 3d). These results showed that ST7L was a direct target for miR-23b.

Furthermore, we measured the expression levels of ST7L in HCC tissues by immunohistochemistry. We divided HCC tissues into two groups with high ST7L expression (score 2 or 3) and low ST7L expression (score 0 or 1). Correlation analysis showed that ST7L expression was inversely correlated with miR-23b expression in HCC tissues (Figures 3e and f). These data suggested that ST7L may be a target of miR-23b in primary HCC tissues as well.

### ST7L is essential for miR-23b-promoted HCC cell proliferation and metastasis *in vitro*

**Figure 4:**
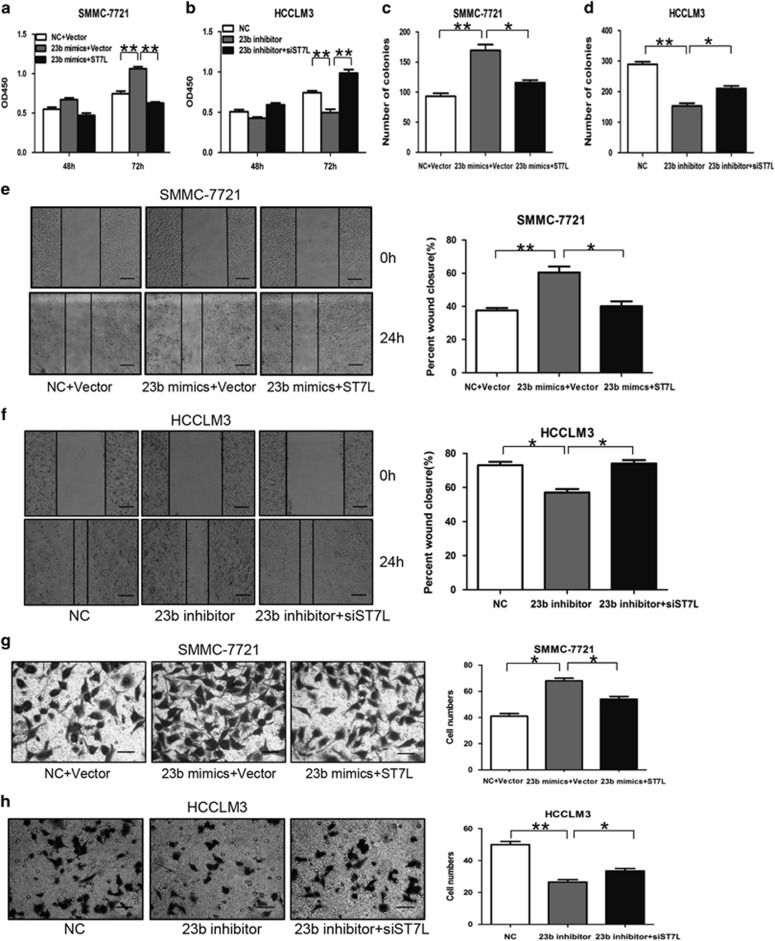
ST7L mediates the effects of miR-23b on proliferation, colony formation and metastasis of HCC cells *in vitro*. SMMC-7721 cells were transiently transfected with miR-23b mimics, ST7L expression plasmids, NC mimics or empty vector, whereas HCCLM3 cells were transfected with miR-23b inhibitor, ST7L siRNA or the corresponding NC oligonucleotides. (**a** and **b**) CCK-8 assays, (**c** and d) colony formation assays, (**e** and **f**) wound-healing assays and (**g** and **h**) transwell invasion assays were performed. The percentage of wound closure and the number of cells passed through the membrane were counted and compared in the right diagrams. Scale bars represent 500 *μ*m (**e** and **f**) and 200 *μ*m (**g** and **h**). Results were represented as mean±S.D. (*n*=3). *P<0.05, **P<0.01

To explore the roles of ST7L in miR-23b-mediated proliferation and metastasis of HCC cells, we transfected ST7L expression plasmids and miR-23b mimics into SMMC-7721 cells. Our results showed that upregulation of ST7L significantly attenuated the promotion effects of miR-23b on proliferation (Figure 4a), colony formation (Figure 4c and Supplementary Figure S1a), migration (Figure 4e) and invasion (Figure 4g) of HCC cells. Accordingly, we transfected ST7L siRNA and miR-23b inhibitor into HCCLM3 cells. Our results showed that downregulation of ST7L obviously abolished the inhibition of miR-23b inhibitor in proliferation (Figure 4b), colony formation (Figure 4d and Supplementary Figure S1b), migration (Figure 4f) and invasion (Figure 4h) of HCC cells. These data demonstrated that miR-23b regulated proliferation and metastasis of HCC cells by targeting ST7L *in vitro*.

### MiR-23b promotes HCC tumor growth and metastasis by targeting ST7L *in vivo*

**Figure 5:**
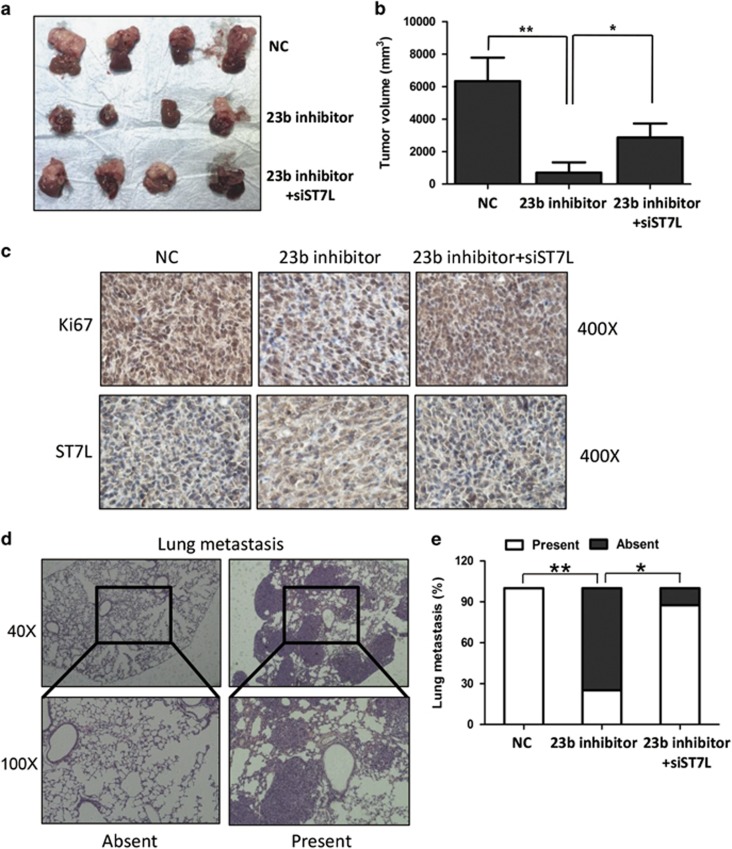
ST7L mediates the effects of miR-23b on HCC progression *in vivo*. HCC mouse model was constructed using stable infected HCCLM3 cells using the corresponding lentiviruses. (**a** and **b**) The sizes of local liver tumors in the three groups were calculated and compared in the right diagrams. Results were represented as mean±S.D. (*n*=4). (**c**) Relative expression levels of Ki67 and ST7L were analyzed by immunohistochemistry. (**d**) Typical pictures for lung metastasis of HCC mouse model. (**e**) The percentage of mice with presence (metastatic lesion⩾1) or absence (metastatic lesion=0) of lung metastasis was calculated (*n*=8). *P<0.05, **P<0.01

To confirm the effects of miR-23b and ST7L on HCC tumorigenesis *in vivo*, we created a xenograft mouse model of HCC. Expression levels of miR-23b in the tumor cells xenografted were shown in Supplementary Figure S2. Consistent with the data *in vitro*, the sizes of tumors generated by HCCLM3 cells infected with miR-23b inhibitor lentivirus were smaller than that of the control group, and knockdown of ST7L attenuated the effects of miR-23b inhibitor (Figures 5a and b). Immunohistochemistry assay showed that the proliferation marker Ki67 was downregulated in the miR-23b knockdown group compared with the control group, and siST7L could abolish the effects of miR-23b knockdown on Ki67 expression (Figure 5c). We also found that expression of ST7L was significantly higher in the group with only miR-23b inhibitor than that in the other two groups (Figure 5c).

To investigate the effects of miR-23b and ST7L on HCC metastasis *in vivo*, we analyzed lung metastasis rates in mouse model (Figure 5d). The incidence of lung metastasis decreased in the miR-23b inhibitor group, and knockdown of ST7L abrogated the effects of miR-23b inhibitor on lung metastasis (Figure 5e). Taken together, these suggested an important role of miR-23b in promoting HCC growth and metastasis *in vivo*.

### MiR-23b regulates AKT/GSK3*β*/*β*-catenin signaling pathway by targeting ST7L in HCC cells

**Figure 6:**
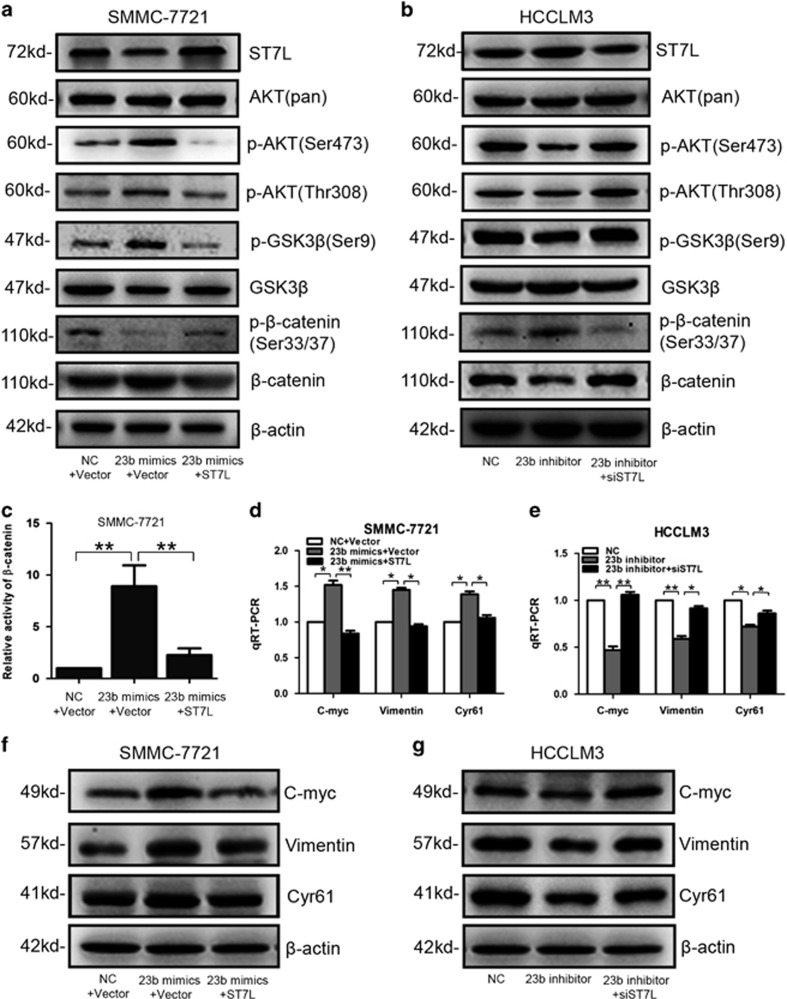
Effects of miR-23b and ST7L on AKT/GSK3*β*/*β*-catenin pathway. SMMC-7721 cells were transiently transfected with miR-23b mimics, ST7L expression plasmids, NC mimics or empty vector, whereas HCCLM3 cells were transfected with miR-23b inhibitor, ST7L siRNA or the corresponding NC oligonucleotides. (**a** and **b**) Western blot analysis of ST7L, AKT (pan), p-AKT (Ser473), p-AKT (Thr308), p-GSK3*β* (Ser9), GSK3*β*, p-*β*-catenin (Ser33/37) and *β*-catenin expression in SMMC-7721 cells or HCCLM3 cells transfected with corresponding plasmids and oligonucleotides. (**c**) Reporter gene analysis of *β*-catenin transactivity in SMMC-7721 cells transfected with corresponding plasmids and oligonucleotides. Data were normalized to the activity of *β*-catenin in SMMC-7721 cells with NC mimics transfection. (**d** and **e**) QRT-PCR analysis of C-myc, Vimentin and Cyr61 expression in SMMC-7721 cells or HCCLM3 cells transfected with corresponding plasmids and oligonucleotides. *β*-Actin was used as a reference gene. (**f** and **g**) Western blot analysis of C-myc, Vimentin and Cyr61 in SMMC-7721 cells or HCCLM3 cells transfected with corresponding plasmids and oligonucleotides. *P<0.05, **P<0.01

To explore the molecular mechanisms through which miR-23b and ST7L regulated tumorigenesis and metastasis of HCC, we examined effector molecules of AKT/GSK3*β*/*β*-catenin signaling pathway, which was related with tumorigenesis and metastasis of many cancers including HCC. Western blot analysis showed that total and nuclear *β*-catenin expression, as well as phosphorylation of AKT (Ser473/Thr308) and GSK3*β* (Ser9) were upregulated by miR-23b, whereas phosphorylation of *β*-catenin (Ser33/37) was downregulated by miR-23b overexpression in SMMC-7721 cells (Figure 6a and Supplementary Figure S3a). We also found that knockdown of miR-23b could decrease total and nuclear *β*-catenin expression, phosphorylation of AKT (Ser473/Thr308) and GSK3*β* (Ser9), and increase phosphorylation (Ser33/37) of *β*-catenin in HCCLM3 cells (Figure 6b and Supplementary Figure S3b). Luciferase analysis showed that miR-23b promoted the transcriptional activity of *β*-catenin (Figure 6c). These data suggested that miR-23b could activate AKT/GSK3*β*/*β*-catenin pathway. We also found that ST7L abolished the effects of miR-23b on the activation of AKT/GSK3*β*/*β*-catenin signaling pathway (Figures 6a–c). Furthermore, our results revealed that miR-23b could increase both mRNA and protein levels of *β*-catenin target genes including C-myc, Vimentin and Cyr61 in HCC cells, whereas ST7L antagonized the effects of miR-23b (Figures 6d–g). All these results suggested that miR-23b could activate the AKT/GSK3*β*/*β*-catenin signaling pathway by suppressing ST7L expression in HCC cells.

### ST7L could interact with AKT and inhibit its activation

**Figure 7:**
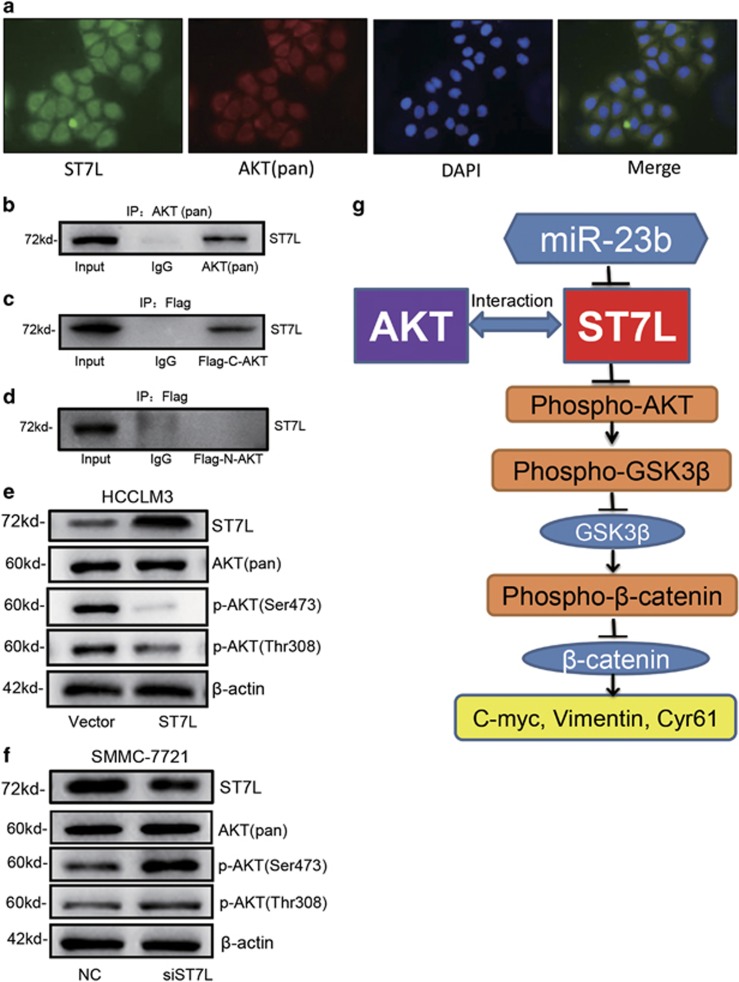
ST7L interacts with AKT and inhibits AKT/GSK3*β*/*β*-catenin pathway in HCC cells. (**a**) Colocalization of ST7L and AKT were detected by immunofluorescence assay in SMMC-7721 cells. ST7L was shown in green, whereas AKT was shown in red. The merge indicated colocalization shown in yellow. (**b**) Co-immunoprecipitation assay for the interaction between ST7L protein and AKT (pan) in SMMC-7721 cells. (**c**) Co-immunoprecipitation assay for the interaction between ST7L protein and C-terminal polypeptides of AKT (409-480aa) in SMMC-7721 cells transfected with flag-tagged C-terminal AKT plasmid. (**d**) Co-immunoprecipitation assay for the interaction between ST7L protein and N-terminal polypeptides of AKT (1–408 aa) in SMMC-7721 cells transfected with flag-tagged N-terminal AKT plasmid. (**e** and **f**) Western blot analysis of ST7L, AKT (pan), p-AKT (Thr308) and p-AKT (Ser473) in HCCLM3 cells with ST7L overexpression and SMMC-7721 cells with ST7L knockdown. (**g**) Summary diagram of the miR-23b/ST7L/AKT pathway in HCC cells

How does ST7L regulate the phosphorylation of AKT and activate AKT/GSK3*β*/*β*-catenin signaling pathway? Maira *et al.*^16^ reported that carboxyl terminal modulator protein (CTMP) bound specifically to the C-terminal domain of AKT and reduced its activity by inhibiting its phosphorylation. Immunofluorescent assay indicated that endogenous AKT and ST7L were colocalized to a large extent in SMMC-7721 cells (Figure 7a). Then, the interaction of endogenous AKT and ST7L were confirmed by co-immunoprecipitation (Figure 7b). Interestingly, we constructed the plasmids expressing N-terminal (amino acids 1–408) or C-terminal (amino acids 409–480) AKT with flag fusion, and found that ST7L could interact with C-terminal but not N-terminal AKT (Figures 7c and d). These results suggested that ST7L may inhibit AKT phosphorylation by binding to C-terminal region of AKT protein. Western blot analysis showed that overexpression of ST7L inhibited phosphorylation of AKT at Ser473 and Thr308 but did not modulate the level of total AKT in HCCLM3 cells (Figure 7e). Accordingly, knockdown of ST7L could increase phosphorylation of AKT in SMMC-7721 cells (Figure 7f).

## Discussion

In the past few years, miRNAs were reported to be involved in tumorigenesis of cancers and function as molecular biomarkers for cancer diagnosis, treatment and prognosis.^17, 18^ Based on small RNA high-throughput sequencing data and many studies focused on the roles of miR-23b in cancers, we explored the roles of miR-23b in HCC. In this study, we found that miR-23b was upregulated in HCC tissues and promoted cell proliferation and metastasis in HCC cell lines and mouse model. Further analysis showed that miR-23b levels were correlated with tumor size, tumor number and BCLC stage in HCC patients. These data suggested that miR-23b may act as a potential diagnostic biomarker and therapeutic target for HCC patients.

MiR-23b exerted various functions in different cancers. In some cancers, miR-23b was reported to function as a tumor-suppressor gene. For instance, miR-23b was downregulated in gastric carcinoma and diminished proliferation and metastasis of gastric cancer cells by inhibiting Notch2 expression.^19^ In bladder cancer, miR-23b was depleted and acted as a tumor-suppressor gene by targeting Zeb1.^20^ Majid *et al.*^21^ also showed that miR-23b repressed Src kinase and inhibit tumorigenesis of prostate cancer. In other cancers, miR-23b was identified as an oncogene. MiR-23b was reported to promote lung metastasis of breast cancer by suppressing the expression of Prosaposin.^8^ Zaman *et al.*^22^ showed that miR-23b was upregulated in renal cancer and induced invasive capability of renal carcinoma cells by targeting PTEN. It seemed that the function of miR-23b in different cancers was determined by the expression levels of miR-23b and specific target genes. MiRNAs could bind to partially complementary sequences in the 3′-UTR of target mRNAs and one miRNA could regulate a series of target genes. In this study, we identified the upregulation of miR-23b in primary HCC tissues via high-throughput sequencing data and qRT-PCR analysis. We also identified the positive effects of miR-23b on HCC cell proliferation and metastasis by targeting ST7L expression. Mir-23a, which was highly homologous to miR-23b, was also reported to be upregulated in HCC tissues and act as an oncogene in HCC progression,^23, 24^ suggesting that these two homologous genes exerted similar expression patterns and functions in HCC. Interestingly, ST7L level was inversely correlated with miR-23b expression in HCC tissues, suggesting that ST7L was an important target gene of miR-23b in HCC tumorigenesis.

ST7L gene, which encodes a polypeptide with 575 amino acids, is located on human chromosome 1p13.^15^ ST7L was homologous to the tumor-suppressor gene ST7 and contained a unique leucine zipper domain. ST7L was reported to be downregulated in glioma and inhibit the tumorigenic behavior of cancer cells.^14^ However, the significance of ST7L in HCC and the underlying mechanism have not been elucidated. Our results showed that miR-23b could interact with 3′-UTR of ST7L and inhibit its expression in HCC cells. Further experiments showed that ST7L could abolish the effects of miR-23b and inhibit cell proliferation and metastasis of HCC cells, suggesting that ST7L acted as a tumor-suppressor gene in HCC progression. Previous study showed that ST7L could inhibit *β*-catenin pathway.^14^ However, the specific mechanism underlying the effects of ST7L was unknown. In this study, our results for the first time verified the interaction between ST7L and AKT, and the effects of ST7L on AKT/GSK3*β*/*β*-catenin signaling pathway.

AKT was frequently activated in cancers and regulated a series of cellular processes. AKT protein contains an N-terminal pleckstrin homology (PH) domain (residues 1–120), a central catalytic domain (residues 121–408) and a C-terminal regulatory domain (residues 409–480).^25^ Phosphorylation of AKT at two sites (Thr308 and Ser473) was critical for AKT activation.^12^ Many proteins were reported to interact with AKT and regulate its activity. For instance, phosphatidylinositol-3, 4, 5-triphosphate bound to PH domain of AKT and induced the phosphorylation of AKT.^26, 27^ CTMP interacted with AKT at the C-terminal region and negatively regulated its activation.^16^ In this study, we revealed the interaction between ST7L and C-terminal region of AKT, which decreased the phosphorylation of AKT and inhibited its activation. These results also suggested an essential role for the C-terminal domain of AKT in its activation.

In summary, we revealed that miR-23b was upregulated in HCC tissues and promoted HCC cell proliferation and metastasis by targeting ST7L *in vitro* and *in vivo*. Our results also showed that miR-23b-mediated ST7L could interact with C-terminal domain of AKT and suppressed AKT/GSK3*β*/*β*-catenin signaling pathway (Figure 7g). Our study not only revealed the important roles of miR-23b and ST7L in HCC progression, miR-23b and ST7L were also the potential diagnostic biomarkers and treatment targets for HCC.

## Materials and Methods

### Tissue specimens of patients

All HCC tissues and ANLTs were randomly collected from patients who underwent liver resection at the Department of Hepatobiliary and Pancreatic Surgery, The Affiliated Hospital of Qingdao University, Qingdao, China with definite pathological diagnosis. Our research strictly conformed to the Ethical Guidelines of the 1975 Declaration of Helsinki, revised in 2000. All human materials were obtained with informed consents and approved by the Ethics Committee of the Affiliated Hospital of Qingdao University.

### Cell culture

In this study, three HCC cell lines (SMMC-7721, SK-Hep1 and HCCLM3) were obtained from Cell Resource Center of Shanghai Institutes for Biological Sciences, Chinese Academy of Sciences (Shanghai, China). Another two HCC cell lines (MHCC97L and MHCC97H) were purchased from Cobioer Biosciences (Nanjing, China). All the cells were cultured in DMEM containing 10% fetal bovine serum (Gibco, Grand Island, NY, USA) in a humidified atmosphere of 5% CO_2_ at 37 °C.

### Quantitative reverse transcription-PCR

Trizol (Invitrogen, Carlsbad, CA, USA) was used to extract total RNA of tissues and cells strictly according to the instructions provided by the manufacturer. Reverse transcription was conducted with Primescript RT Master Mix (Takara, Otsu, Japan), and cDNA was amplified using SYBR-Green Premix (Takara). RNU66 and U6 were used as reference genes for miR-23b in tissues and cells, respectively. MiR-23b and RNU66 expression levels were detected using Hairpin-itTM Quantitation PCR Kit (GenePharma, Shanghai, China). The data were analyzed by delta Ct method. Primers used in this study were listed in Supplementary Table S1.

### Small RNA sequencing

Total RNAs were extracted from eight HCC tissues and eight ANLTs, and sent to Beijing Genomics Institute (Shenzhen, China) for sequencing of small RNAs. Expression profile of small RNAs was sorted using Cluster 3.0 software (University of Tokyo, Human Genome Center, Tokyo, Japan).

### Transfection and luciferase reporter assay

MiR-23b mimics, miR-23b inhibitor, ST7L siRNA and NC oligonucleotides were obtained from GenePharma. ST7L expression plasmids and flag-tagged AKT plasmids were obtained from Biogot Technology (Nanjing, China). To generate reporter construct, one fragment of ST7L 3′-UTR including two putative miR-23b complementary sites was fused to a modified pcDNA3.1 vector containing a luciferase gene, which was inserted upstream of multiple cloning sites. Reporter plasmids with mutant sites were prepared by Mutagenesis Kit (Stratagene, La Jolla, CA, USA). TOP/FLASH reporter gene including *β*-catenin binding sites was obtained from Millipore (Billerica, MA, USA). Oligonucleotides were transfected by Hiperfect transfection reagent (Qiagen, Valencia, CA, USA) and plasmids were transfected by Lipofectamine 3000 (Invitrogen) into cells. Luciferase activity assay was conducted using Dual Luciferase Assay System (Promega, Madison, WI, USA).

### Cell proliferation assays

CCK-8 (Dojindo, Kumamoto, Japan) assay was performed to evaluate cell proliferation. Corresponding oligonucleotides or plasmids were transfected into cells, which were seeded in a 96-well plate and cultured at 37 °C. At various time points, CCK-8 was added into each well of the plate. Absorbance at 450 nm was measured after 30-min incubation at 37 °C.

### Colony formation assays

Cells were transfected with corresponding oligonucleotides or plasmids, and cultured at 37 °C for 24 h. Then, cells were digested with 0.25% trypsin and made into single-cell suspensions with DMEM. Cell number was counted with Scepter 2.0 Handheld Automated Cell Counter (Millipore). In all, 500 cells per well were seeded in a six-well plate. After about 2 weeks of culture, the colonies were fixed using methanol, dyed with 5% crystal violet and counted.

### Wound-healing assays

Cells were transfected with corresponding oligonucleotides or plasmids, and cultured at 37 °C for 24 h before wounds were created with a 10 *μ*l pipette tip in a 12-well plate. Then, cells were cultured in DMEM without fetal bovine serum at 37 °C. Images were taken at 0 h and 24 h after wounds were created, and Image J software (NIH, Bethesda, MD, USA) was used to analyze the results.

### Transwell invasion assays

Cell invasion assays were performed using 24-well transwell chambers with 8.0*μ*m pore size polycarbonate membrane (Corning Incorporated, Corning, NY, USA). Cells were transfected with corresponding oligonucleotides or plasmids, and cultured at 37 °C for 24 h. Then, cells were digested and seeded with Matrigel (BD, Franklin Lakes, NJ, USA), and cultured with DMEM without fetal bovine serum in the upper chamber. In the lower 24-well plate, DMEM containing 10% fetal bovine serum was added. After appropriate time (24 h for SMMC-7721 cells and 12 h for HCCLM3 cells), cells inside the upper chamber were removed with cottons swabs. Cells invaded through the membrane surface were fixed with methanol, stained with 5% crystal violet and counted.

### Western blot

Total protein was extracted, separated by sodium dodecyl sulfate-polyacrylamide gel electrophoresis and transferred onto 0.45*μ*m PVDF membranes (Millipore). The following primary antibodies were used in the immunoblotting assays: antibodies for ST7L (17567-1-AP; Proteintech, Wuhan, China), Vimentin (no.3390; Cell Signaling Technology, Danvers, MA, USA), *β*-actin (no.3700; Cell Signaling Technology), C-myc (ab17356; Abcam, Cambridge, MA, USA), AKT (pan) (no.4691; Cell Signaling Technology), phospho-AKT (Ser473) (no. 4060; Cell Signaling Technology), phospho-AKT (Thr308) (no. 13038; Cell Signaling Technology), phospho-*β*-catenin (Ser33/37) (AP3908a; Abgent, WuXi, China), Cyr61 (no. 14479; Cell Signaling Technology), GSK3*β* (no.12456; Cell Signaling Technology), phospho-GSK3*β* (Ser9) (no. 14479; Cell Signaling Technology), *β*-catenin (no.8480; Cell Signaling Technology) and TBP (ab125009; Abcam).

### Immunohistochemistry

Immunohistochemistry was performed on paraformaldehyde-fixed paraffin sections. ST7L antibody (sc-138649; Santa Cruz, Biotechnology, Santa Cruz, CA, USA) and Ki67 antibody (bs-23103R, BIOSS, Beijing, China) were used in immunohistochemistry with streptavidin peroxidase-conjugated method. The percentage of positive cells was graded as the following criteria: 0, below 10% 1, 10–30% 2, 31–50% 3, above 50%.

### Immunofluorescence

Cells cultured on glass coverslips were fixed with 4% paraformaldehyde, blocked with 5% bovine serum albumin and incubated with AKT (pan) antibody (no.4691; Cell Signaling Technology) and ST7L antibody (sc-138649; Santa Cruz, Biotechnology) overnight at 4 °C. Then, cells were incubated with mouse anti-goat IgG/FITC antibody (bs-0294M-FITC, BIOSS) and goat anti-rabbit IgG/Cy3 antibody (bs-0295G-Cy3, BIOSS) for 2 h at room temperature. DAPI (Invitrogen) was used to stain the nucleus. LEICA DMI4000B microscope (Leica, Heidelberg, Germany) was used to take the images.

### HCC xenograft mouse model

The BALB/c nude mice were obtained from the Shanghai Institute of Materia Medica (Shanghai, China). Stable infected HCCLM3 cells (5 × 10^6^), which were previously infected with miR-23b or ST7L knockdown lentivirus were injected subcutaneously to the posterior flank of the nude mice. One week later, the subcutaneous tumors were removed and implanted into the livers of new nude mice. Five weeks later, mice were killed. Tumor volumes were measured and the size was calculated as follows: tumor volume (mm^3^)=(length × width^2^)/2. The lung of mice were sectioned and stained with hematoxylin to identify metastatic lesions. All animal studies we performed were conducted in the Animal Institute of Affiliated Hospital of Qingdao University according to the protocols approved by the Medical Experimental Animal Care Commission of Qingdao University.

### Co-immunoprecipitation assay

Total protein lysates were extracted with NP-40 buffer (BOSTER, Wuhan, China). Extracted protein and Protein A/G Plus-Agarose (sc-2003, Santa Cruz, Biotechnology) were mixed with normal IgG (Beyotime, Shanghai, China) or indicated antibody at 4 °C overnight. Then, the precipitated proteins were boiled in SDS-loading buffer (4% SDS, 10% glycerol, 5% *β*-mercaptoethanol, 100 mmol/l Tris (pH 6.8)) and analyzed by western blot. The following primary antibodies were used: AKT (pan) (no.4691; Cell Signaling Technology), ST7L (17567-1-AP; Proteintech) and Flag (66008-2-Ig; Proteintech).

### Statistical analysis

Statistical analysis was performed using the SPSS program (version 13.0; SPSS, Chicago, IL, USA). The statistical significance of differences between two groups was calculated by Student's *t*-test or *χ*^2^ test. Multiple comparisons were performed using the one-way ANOVA followed by Newman–Keuls test. Spearman's analysis was used in correlation analysis. *P*<0.05 was considered as statistically significant.

## Figures and Tables

**Table 1:** Correlation between the clinicopathologic characteristics and miR-23b expression in HCC tissues

**Factor**	**Total number**	***n***		***P*-value**
		**MiR-23b high expression**	**MiR-23b low expression**	
*Gender*				
Male	60	39	21	
Female	10	5	5	0.285

*Age (years)*				
⩽60	51	34	17	
>60	19	10	9	0.210

*Tumor size (cm)*				
⩽5	48	26	22	
>5	22	18	4	**0.023**

*Tumor number*				
1	53	30	23	
⩾2	17	14	3	**0.049**

*AFP (ng/ml)*				
⩽20	30	20	10	
>20	40	20	20	0.375

*ALT (U/l)*				
⩽40	52	33	19	
>40	18	11	7	0.537

*BCLC stage*				
0–A	53	29	24	
B–C	17	15	2	**0.011**

*Liver capsule invasion*				
No	66	41	25	
Yes	4	3	1	0.524

*Liver cirrhosis*				
Absent	3	2	1	
Present	67	42	25	0.691

*Hepatitis B status*				
Negative	5	3	2	
Positive	65	41	24	0.619

Abbreviations: AFP, *α*-fetoprotein; ANLT, adjacent non-tumor liver tissue; BCLC, Barcelona Clinic Liver Cancer; HCC, hepatocellular carcinoma

MiR-23b high expression: expression of miR-23b in HCC tissues was higher than that in corresponding ANLTs; miR-23b low expression: expression of miR-23b in HCC tissues was lower than that in corresponding ANLTs. Bold numbers indicate significant differences (*P<*0.05)
