# Supplementary material for: MicroRNA-23b functions as an oncogene and activates AKT/GSK3β/β-catenin signaling by targeting ST7L in hepatocellular carcinoma
Source: Cell Death Dis. 2017 May 18;8(5):e2804–. doi: 10.1038/cddis.2017.216 (PMC5520730; doi:10.1038/cddis.2017.216)
Supplement: Supplementary Table S1 [file cddis2017216x1.docx]

**Table S1.** Primers used in this study.

| **Primer** | **Sequences** |
| --- | --- |
| C-myc Forward primer | CAAGAGGCGAACACACAACGT |
| C-myc Reverse primer | GGGCCTTTTCATTGTTTTCCA |
| Vimentin Forward primer | GAGAACTTTGCCGTTGAAGC |
| Vimentin Reverse primer | GCTTCCTGTAGGTGGCAATC |
| Cyr61 Forward primer | GCAGTTGGAAAAGGCAGCTC |
| Cyr61 Reverse primer | ACAGGTCTTTGAGCACTGGG |
| β-actin Forward primer | CATCCTCACCCTGAAGTACCCC |
| β-actin Reverse primer | AGCCTGGATGCAACGTACATG |
| U6 Forward primer | TCGCTTCGGCAGCACATA |
| U6 Reverse primer | TTTGCGTGTCATCCTTGC |
