# Supplementary figures and images for: MicroRNA-23b functions as an oncogene and activates AKT/GSK3β/β-catenin signaling by targeting ST7L in hepatocellular carcinoma
Source: Cell Death Dis. 2017 May 18;8(5):e2804–. doi: 10.1038/cddis.2017.216 (PMC5520730; doi:10.1038/cddis.2017.216)

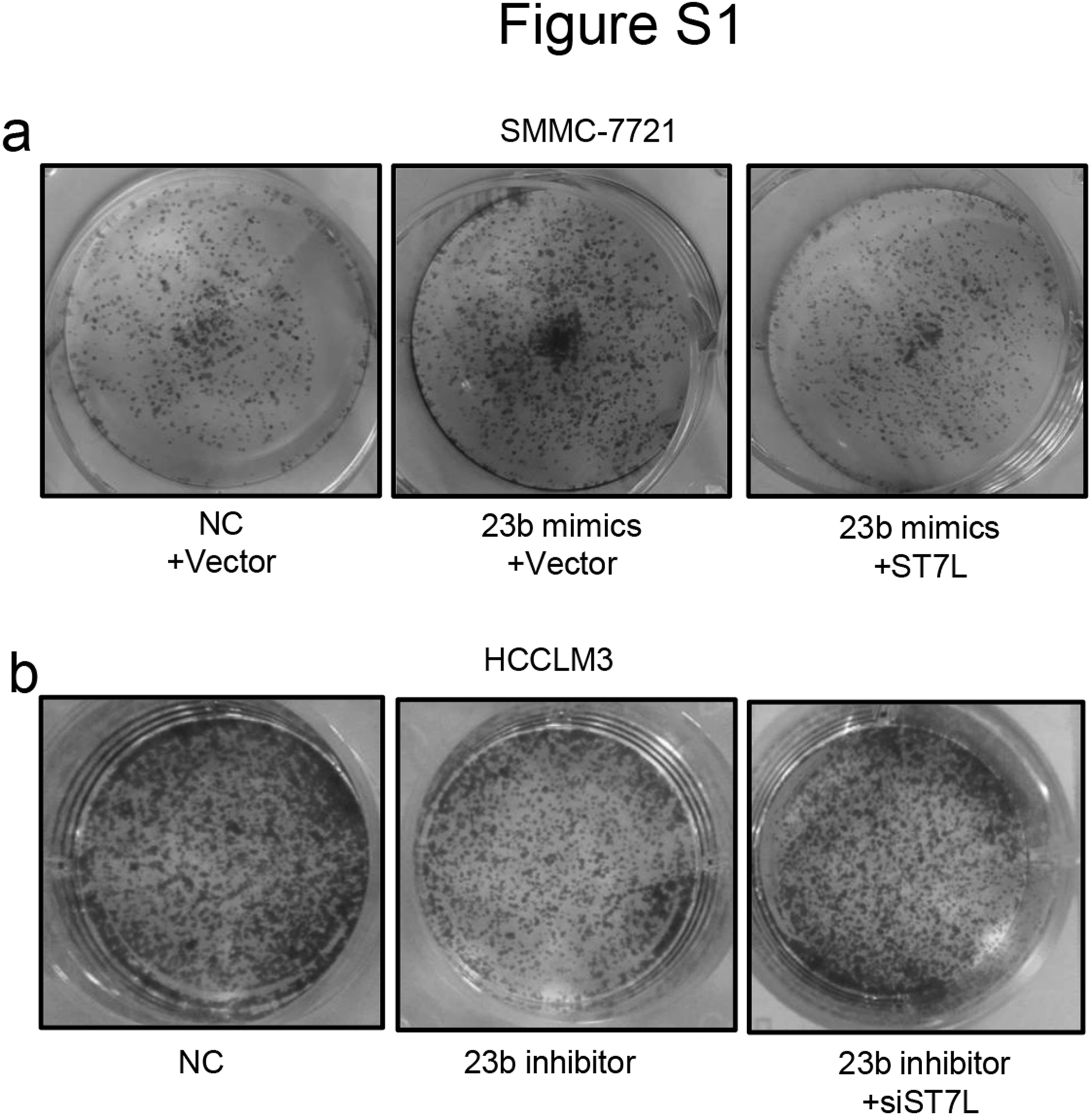

Supplement: Supplementary Figure S1 [file cddis2017216x2.tif]

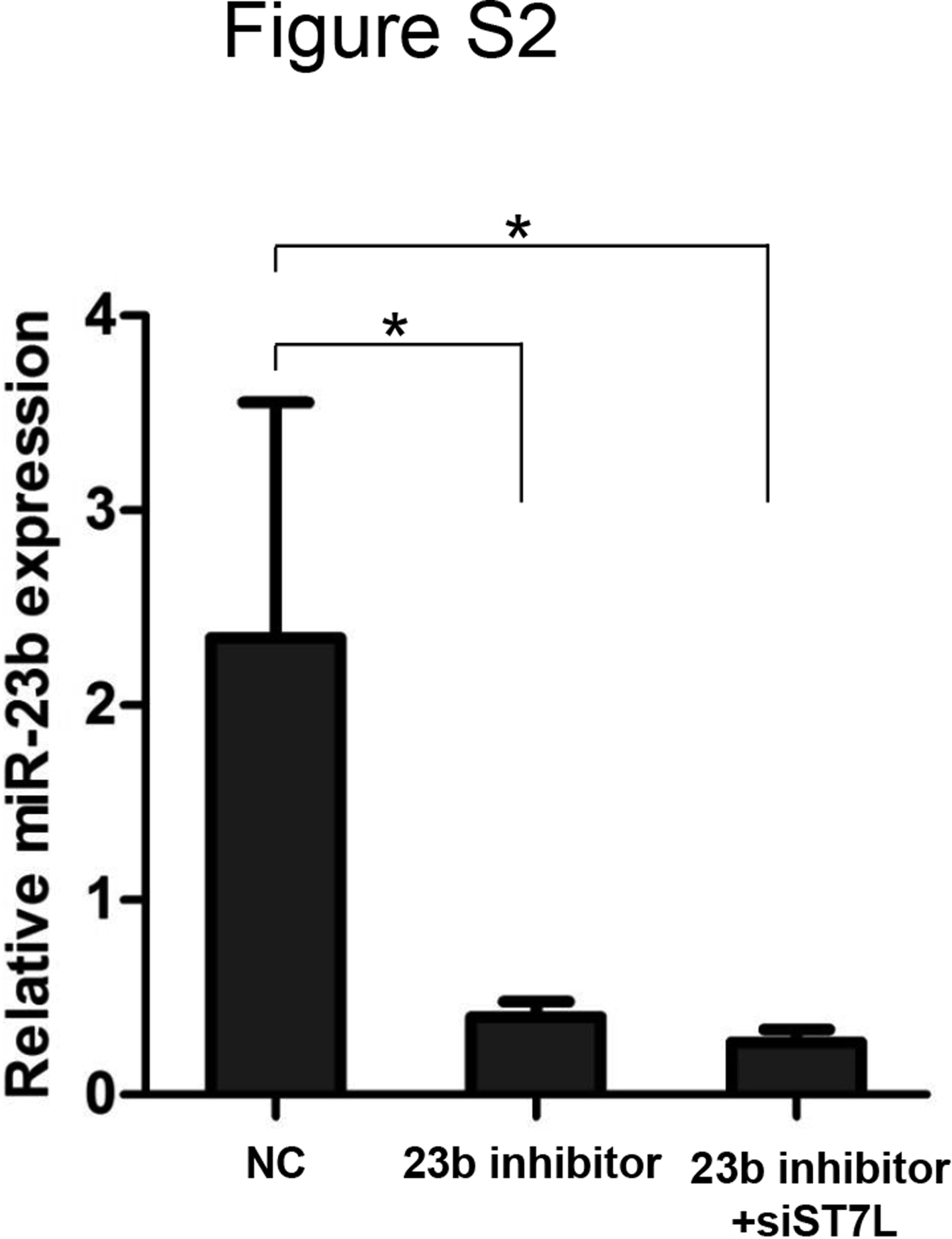

Supplement: Supplementary Figure S2 [file cddis2017216x3.tif]

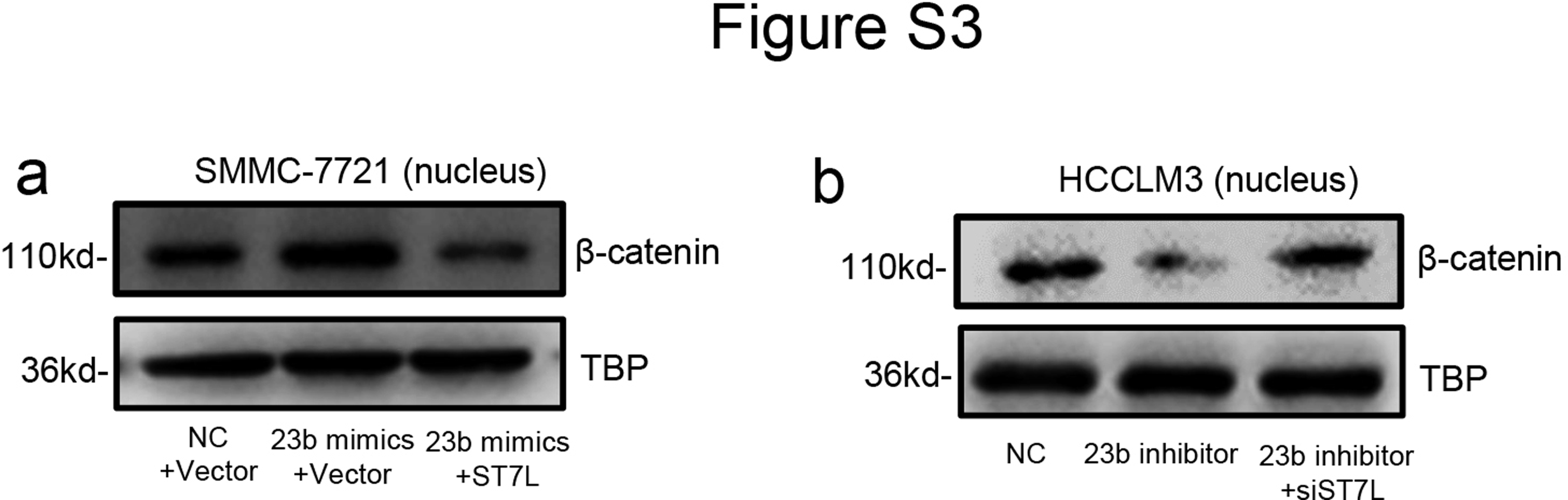

Supplement: Supplementary Figure S3 [file cddis2017216x4.tif]
