# Supplementary material for: MicroRNA-23b functions as an oncogene and activates AKT/GSK3β/β-catenin signaling by targeting ST7L in hepatocellular carcinoma
Source: Cell Death Dis. 2017 May 18;8(5):e2804–. doi: 10.1038/cddis.2017.216 (PMC5520730; doi:10.1038/cddis.2017.216)
Supplement: Supplementary Figure Legends [file cddis2017216x5.docx]

**Suplplementary Figure legends**

**Figure S1.** Representative images of colony formation assays in SMMC-7721 cells transfected with miR-23b mimics and ST7L-expressing plasmids (a), and HCCLM3 cells transfected with miR-23b inhibitor and ST7L siRNA (b).

**Figure S2.** QRT-PCR analysis of miR-23b expression in xenograft tumors generated by stable infected HCCLM3 cells. Data were normalized to U6 expression. Results were represented as mean ± S.D. (n =4). **P* < 0.05

**Figure S3.** Nuclear β-catenin protein in SMMC-7721 cells transfected with miR-23b mimics and ST7L-expressing plasmids (a) and HCCLM3 cells transfected with miR-23b inhibitor and ST7L siRNA (b).
